# Patterns of physical activity and sedentary behaviour in preschool children

**DOI:** 10.1186/1479-5868-9-138

**Published:** 2012-11-27

**Authors:** Eveline Van Cauwenberghe, Rachel A Jones, Trina Hinkley, David Crawford, Anthony D Okely

**Affiliations:** 1 Department of Movement and Sport Sciences, University of Ghent, Watersportlaan 2, Ghent, Belgium; 2 Interdisciplinary Educational Research Institute, University of Wollongong, Wollongong, NSW, 2522, Australia; 3 Centre for Physical Activity and Nutrition Research, Deakin University, Burwood, Victoria, 3125, Australia

**Keywords:** Accelerometry, Physical activity, Sedentary behavior, Variability, Hour-by-hour, Young children

## Abstract

**Background:**

Little is known about patterns of sedentary behavior (SB) and physical activity among preschoolers. Therefore, in this observational study patterns of SB and moderate-to-vigorous physical activity (MVPA) were examined in detail throughout the week in preschool-aged boys and girls.

**Methods:**

A sample of 703 Melbourne preschool children (387 boys; 4.6 ± 0.7 y) were included in data analysis. SB and MVPA data were collected using accelerometry over an eight-day period. Percentage of time per hour in SB and in MVPA between 08:00 h and 20:00 h was calculated. Multi-level logistic regression models were created to examine the hour-by-hour variability in SB and MVPA for boys and girls across weekdays and weekend days. Odds ratios (OR) were calculated to interpret differences in hour-by-hour SB and MVPA levels between boys and girls, and between weekdays and weekend days.

**Results:**

The highest SB levels co-occurred with the lowest MVPA levels from the morning till the early afternoon on weekdays, and during the morning and around midday on weekends. Besides, participation in SB was the lowest and participation in MVPA was the highest from the mid afternoon till the evening on weekdays and weekend days. The variability across the hours in SB and, especially, in MVPA was rather small throughout weekdays and weekends. These patterns were found in both boys and girls. During some hours, girls were found to be more likely than boys to demonstrate higher SB levels (OR from 1.08 to 1.16; all p < 0.05) and lower MVPA levels (OR from 0.75 to 0.88; all p < 0.05), but differences were small. During weekends, hour-by-hour SB levels were more likely to be lower (OR from 0.74 to 0.98; all p < 0.05) and hour-by-hour MVPA levels were more likely to be higher (OR from 1.15 to 1.50; all p < 0.05), than during weekdays, in boys and girls.

**Conclusion:**

Entire weekdays, especially from the morning till the early afternoon, and entire weekend days are opportunities to reduce SB and to promote MVPA in preschool-aged boys and girls. Particularly weekdays hold the greatest promise for improving SB and MVPA. No particular time of the week was found where one sex should be targeted.

## Background

The preschool years (between 3 and 5 years of age) represent one of the critical periods in which health behaviors, such as physical activity (PA) and sedentary behavior (SB), are established [1]. Regular PA during the preschool years protects against the accumulation of excess body fat [2], while high levels of SB are linked with an increased risk of being overweight or obese [3]. Furthermore, regular PA aids in the motor, musculoskeletal, social, and psychological development of preschool children [4]. Several countries have developed recommendations that preschoolers should participate in at least several hours of PA of any intensity and limit the time spent being sedentary, particularly in screen-based entertainment such as television viewing and electronic games [5-7]. Despite their importance, recent reviews, summarizing the international evidence on objectively measured levels of habitual PA and SB, demonstrate that preschool children generally exhibit low levels of PA and high levels of SB [8,9]. The preschool period may, therefore, provide a valuable opportunity for the promotion of PA and the reduction of SB.

Accelerometers are currently considered to be the method of choice for measuring free-living PA and SB in preschool children [10,11]. Accelerometers are appropriate to use in preschoolers, enable objective quantification and interpretation of the frequency, intensity, and duration of PA during a total day or segmented parts of the day, across several days, and allow investigations in large samples [10,11]. Although the number of accelerometer-based studies in preschoolers has increased in recent years, there are still several areas where research is needed [8,12,13]. For example, accelerometer-based studies that provide detailed knowledge of the days of the week and specific periods within a day in which low engagement in PA and high participation in SB are typical for preschool children may facilitate the identification of intervention opportunities and may increase our understanding of PA and SB in preschoolers. To date, accelerometer-based studies in preschoolers have examined average daily PA levels during weekdays and weekends [14-22] or described average PA levels during specific time periods of the day, such as recess and out-of-home care [23-25]. Despite the ability of accelerometers to investigate in detail PA throughout the day, only four studies have reported on PA variability in preschoolers across different segments of the day, for example in-preschool versus out-of-preschool [14,16,18,21], and only one study in preschoolers has reported on PA patterns hour-by-hour [20]. Hour-by-hour patterning of activity engagement throughout a day is important because it can provide information on when preschool children are engaging in more or less PA and SB, thus potentially highlighting critical windows to intervene. Moreover, in most of these previous studies, PA was reported as total PA (expressed as counts per minute or activity energy expenditure) or time spent in moderate-to-vigorous physical activity (MVPA). Only four have reported time spent in SB and/or light intensity PA [15,16,22,25]. A limited number of these studies were conducted in medium to large sample sizes (more than 250 participants) with none involving more than 800 participants [16,23,24]. Furthermore, although differences in behavior between boys and girls were mostly examined, none stratified by activity level or weight status, potentially missing key times for subgroups of children.

The purpose of this observational study following the STROBE guidelines [26] was, therefore, to examine patterns of accelerometer-based SB and MVPA hour-by-hour in a large sample of preschool-aged children. SB and MVPA were evaluated to gain more insight in preschoolers’ patterns of inactive and high intense activities. Patterns of total PA (i.e., PA of any intensity, namely light-to-vigorous PA) were not examined since they are just the inverse of the SB patterns. Given that differences have been found between boys and girls in SB and PA [12,27] and weekdays are structured differently from weekend days, patterns were examined separately in boys and girls and for weekdays and weekend days. Further, patterns were investigated by SB and MVPA level and by weight status to identify if patterns differed across these subgroups of children.

## Methods

### Recruitment and participants

Methods of the current study have been published previously [28,29]. Baseline data were drawn from the Healthy Active Preschool Years (HAPPY) study, a cohort study which investigated correlates of PA and SB in preschoolers. The study was approved by the Deakin University Human Research Ethics Committee and the Victorian Department of Education and Early Childhood Development. Childcare centers and preschools located in two low, two medium, and two high socio-economic position areas of metropolitan Melbourne, Australia, were targeted with the final sample involving 71 childcare centers (46% of those approached) and 65 preschools (47% of those approached). Parents of all children aged 3 to 5 years (n = 9794) were invited to participate in the study via written information letters and consent forms. Of this number, 1036 parents (11%) consented for their children to participate. Of these participants, four children were six years old at the time of data collection and 28 parents withdrew before the start of the data collection, resulting in a final sample of 1004 preschoolers. Recruitment and data collection occurred in two phases, between July and November 2008 and June and October 2009. These data collection periods covered winter (average minimum and maximum temperature of 8°C and 15°C, respectively) and spring (average minimum and maximum temperature of 10°C and 23°C, respectively). Measures relevant to this study were accelerometry, demographics, anthropometrics, and parental reported attendance times and days at child care/preschool of the child [29].

### Accelerometry

On the first day of the protocol, participants were fitted with a GT1M ActiGraph accelerometer at childcare/preschool by a trained researcher. These uniaxial accelerometers, designed to detect vertical accelerations, have established utility, validity, and reliability in preschool-aged children [10]. Accelerometers were worn on an elastic belt around the waist and positioned on the right hip. Participants wore the accelerometer during eight consecutive days during waking hours. Parents of participants were instructed to remove the accelerometer for sleeping/napping time and aquatic activities such as swimming and bathing (i.e., the monitor was not worn during these times). Accelerometers commenced recording at 09:00 h (09:00 am) on the day of fitting and data were collected in 15 second epochs. On or after the eighth day of the protocol, accelerometers were collected by the researcher at childcare/preschool. Data were downloaded and raw data files were then reduced using the software Meterplus version 4.2 (Santech Inc., San Diego, US). In accordance with previous accelerometer studies in preschool children, periods containing 10 minutes or more of consecutive zero counts were deleted, as these periods were regarded as non-wearing time [30,31]. Additionally, days with a wear time higher than 18 hours [32] and lower than 50% of the child’s usual wake time (390 ± 32 min; range: 240 – 540 min) were excluded [29]. The child’s usual wake time was calculated by subtracting the parent-reported child’s usual night and day sleep time from 1440 minutes (24 hours * 60 minutes).

Participants were included for analyses if they had a minimum of three weekdays and one weekend day of data [29,33]. For the purpose of this study, the proportion of time per hour (reported as percentage for ease of understanding) in SB and in MVPA were calculated. The hours between 08:00 h and 20:00 h were included as at least 75% of participants recorded data during these times. To estimate SB, the following cut point was applied: ≤ 25 counts/15 s [34]. This cut point has been shown to provide the least bias in the estimation of SB among the currently published [35,36] and is widely applied in large national surveys [37,38]. To define MVPA, a very common cut point [29,30,39] for this age group was applied: > 614 counts/15 s for 3-year olds, > 811 counts/15 s for 4-year olds, and > 890 counts/15 s for 5-year olds [40]. Further, tertiles of the mean percentage of time per day in SB and MVPA were calculated for boys and girls separately. Based on these tertiles, boys and girls with low (lowest SB tertile) and high (highest SB tertile) SB levels and boys and girls with low (lowest MVPA tertile) and high (highest MVPA tertile) MVPA levels were identified.

### Demographics and anthropometrics

On the day the accelerometer was fitted, preschooler’s height and weight were measured by the researcher and a survey was provided to be completed by the parents. After participants removed their shoes and were in light clothing, height was measured to the nearest 0.1 cm using a Wedderburn Seca portable rigid stadiometer. Weight was measured to the nearest 0.1 kg using a Wedderburn Tanita portable digital scale. The average of two measures was recorded. If the two measures differed by more than 0.5 cm and 0.5 kg, respectively, a third measure was taken and the average of the two closest measures was used. Child’s body mass index (BMI; kg^.^m^-2^) was calculated and child’s weight status was determined using the age- and sex-specific BMI thresholds of Cole and colleagues [41]. Preschooler’s demographics (sex and date of birth) were acquired through the parental survey.

### Statistical analyses

To examine patterns of percentage of time in SB and MVPA hour-by-hour, a series of multi-level logistic regression models for binomial response data with a logit link function were conducted using MLwiN 2.23 (Centre for Multilevel Modelling, University of Bristol, UK). Variability in percentage of time per hour in SB and MVPA across the included hours (08:00 h to 20:00 h) for boys and girls during weekdays and weekends was investigated by adding 11 dummy variables (i.e., time 09:00 h to 20:00 h was contrasted with time 08:00 h) to the model. When the main effect of time showed statistical significance, hour-by-hour variability was tested by changing the reference category (by default 08:00 h). Differences in percentages of time per hour in SB and MVPA according to sex (dummy variable: boys vs. girls) during weekdays and weekends were also examined. Further, differences in percentages of time per hour in SB and MVPA according to type of day (dummy variable: weekdays vs. weekend days) in boys and girls were studied. Finally, to establish whether the hour-by-hour patterns differed by SB and MVPA level (dummy variable: low vs. high) and by weight status (dummy variable: healthy weight vs. overweight/obese), interaction effects were examined within these models. All analyses controlled for the nested structure of the data: hour-by-hour accelerometer measurements across several days nested within participants and participants nested within centre of recruitment. Although participants’ SB and MVPA data were not available for all hours and for all days (because of not wearing the accelerometer), the occurrence of missing data does not constitute a problem since the software automatically takes missing data into account [42]. To determine the expected percentages of time per hour in SB and MVPA for boys and girls during weekdays and weekends, the predicted logit values were transformed back using the logistic function g(β) = e^β^/ (1 + e^β^) [42]. To interpret the differences in percentages of time per hour in SB and MVPA between boys and girls and between weekdays and weekends, odds ratios (OR) were calculated by exponentiating the logistic coefficient: e^β^[42]. Wald tests [42] were used to test statistical significance at the alpha level of 0.05.

## Results

### Participants

One accelerometer recording day was lost because of a wearing time greater than 18 hours while 3016 days were lost because of a wearing time below 50% of the child’s wake time, resulting in a total of 703 preschoolers (70% of those recruited) with sufficient accelerometer data. Descriptive sample characteristics are shown in Table 1 and the sample has been described in more detail in a previously published paper [28]. The participants with sufficient accelerometer data did not vary from the participants with non-sufficient accelerometer data by gender, age, BMI, and weight status (all p > 0.05). In the analysis sample, accelerometers were worn on average for 646 (± 122) minutes per weekday and 660 (± 114) minutes per weekend day in boys and for 640 (± 116) minutes per weekday and for 656 (± 112) minutes per weekend day in girls. On average 1.9 ± 0.4 weekend days and 5.0 ± 0.8 weekdays per preschooler were included for analysis. Boys and girls accumulated on average 306 (± 77) and 309 (± 70) minutes in SB, respectively, during weekdays and 293 (± 23) and 300 (± 78) minutes in SB, respectively, during weekends. Time spent in MVPA for boys and girls was 32 (± 21) and 27 (± 17) minutes, respectively, during weekdays and 36 (± 25) and 30 (± 21) minutes, respectively, in MVPA during weekends. According to the parental reports, children attended child care/preschool for 1 to 5 weekdays (median: 3 weekdays; IQR: 2 to 3 weekdays) per week and for 1 to 11 hours per weekday (median: 6 hours; IQR: 3 to 8 hours). Median start attendance time was 09:00 h (IQR: 08:30 h to 09:15 h; range: 06:00 h to 14:00 h); median end attendance time was 15:45 h (IQR: 14:00 h to 16:30 h; range: 09:00 h to 18:30 h).

**Table 1:** Descriptive characteristics of participants

**Descriptive characteristics**	**Total sample (n=703)**	**Boys (n=387)**	**Girls (n=316)**
Age (y)	4.6±0.7	4.6±0.7	4.6±0.7
Age group (%)			
- 3.0 to 3.9 y olds	24.3	23.8	25.0
- 4.0 to 4.9 y olds	43.1	43.1	43.0
- 5.0 to 5.9 y olds	32.6	33.1	32.0
Height (cm) ^*^	107.5±6.6	108.0±6.5	106.9±6.8
Weight (kg) ^**^	18.9±3.0	19.1±2.8	18.7±3.2
BMI (kg^.^m^-2^) ^**^	16.3±1.5	16.3±1.3	16.2±1.6
Weight status (%) ^**^			
- Healthy weight	82.5	84.4	80.3
- Overweight/ obese	17.5	15.6	19.7

^*^: 3 missing values; ^**^: 4 missing values.

### SB patterns hour-by-hour

**Figure 1:**
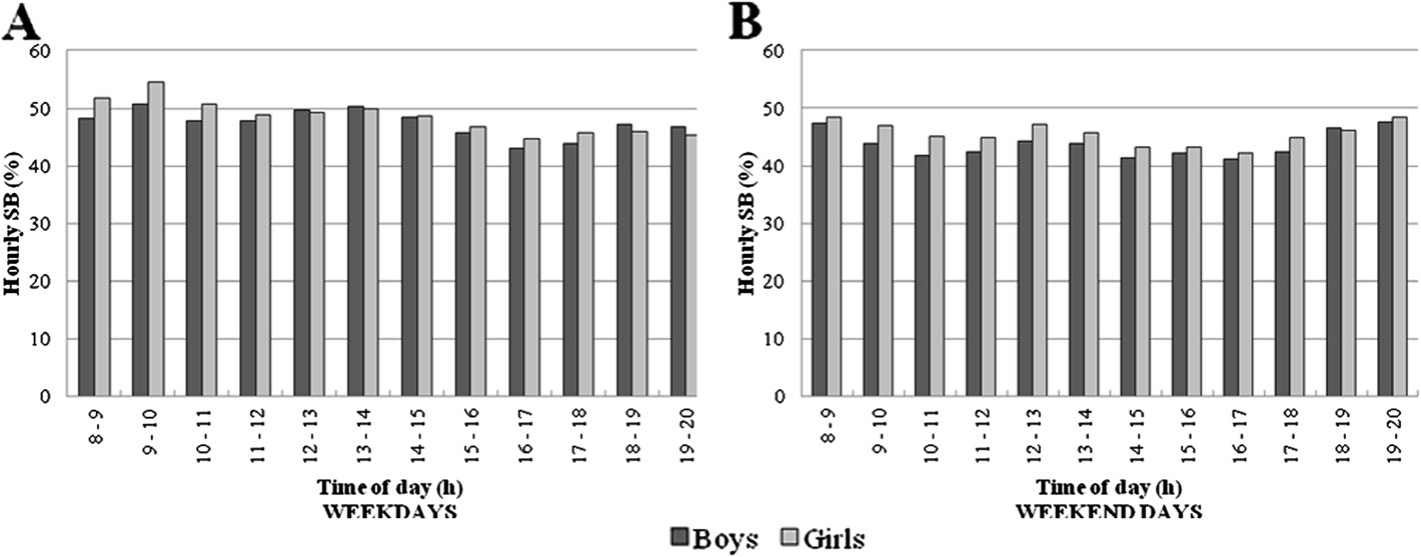
SB patterns hour-by-hour during weekdays and weekend days for boys and girls.

During weekdays, the percentage of time in SB varied significantly hour-by-hour in boys (χ^2^ (Δ 11 df) = 310.8) and girls (χ^2^ (Δ 11 df) = 432.9) (Figure 1A). The highest percentages of time per hour in SB for boys were found from 09:00 h to 10:00 h and 12:00 h to 14:00 h while girls were most sedentary from 08:00 h to 11:00 h. After 14:00 h for boys and after 11:00 h for girls, the percentages of time per hour in SB declined. The lowest percentages of time per hour in SB were found in boys from 15:00 h to 18:00 h and in girls from 15:00 h to 20:00 h. From 08:00 h to 11:00 h and 17:00 h to 18:00 h, girls were significantly more likely than boys to demonstrate a higher percentage of time per hour in SB (OR ranging from 1.08 to 1.15).

During weekend days, the percentage of time in SB varied significantly hour-by-hour in boys (χ^2^ (Δ 11 df) = 110.7) and girls (χ^2^ (Δ 11 df) = 79.6) (Figure 1B). The percentage of time per hour in SB peaked between 08:00 h and 10:00 h, 12:00 h and 14:00 h, and 18:00 h and 20:00 h in both boys and girls. In boys, the lowest percentages of time in SB were observed from 10:00 h to 11:00 h, 14:00 h to 15:00 h, and 16:00 h to 17:00 h. In girls, the lowest percentages of time in SB were seen from 14:00 h to 17:00 h. Between 09:00 h and 13:00 h and 17:00 h to 18:00 h, it was significantly more likely to find a higher percentage of time per hour in SB in girls compared to boys (OR ranging from 1.11 to 1.16).

For boys and girls, the percentage of time per hour in SB was significantly more likely to be lower during weekends from 08:00 h till 17:00 h compared to weekdays (OR ranging from 0.76 to 0.98 and 0.74 to 0.93 in boys and girls, respectively). In girls, percentage of time per hour in SB was significantly more likely to be higher during weekends from 19:00 h to 20:00 h than during weekdays (OR = 1.10).

### SB patterns by low and high SB levels

**Figure 2:**
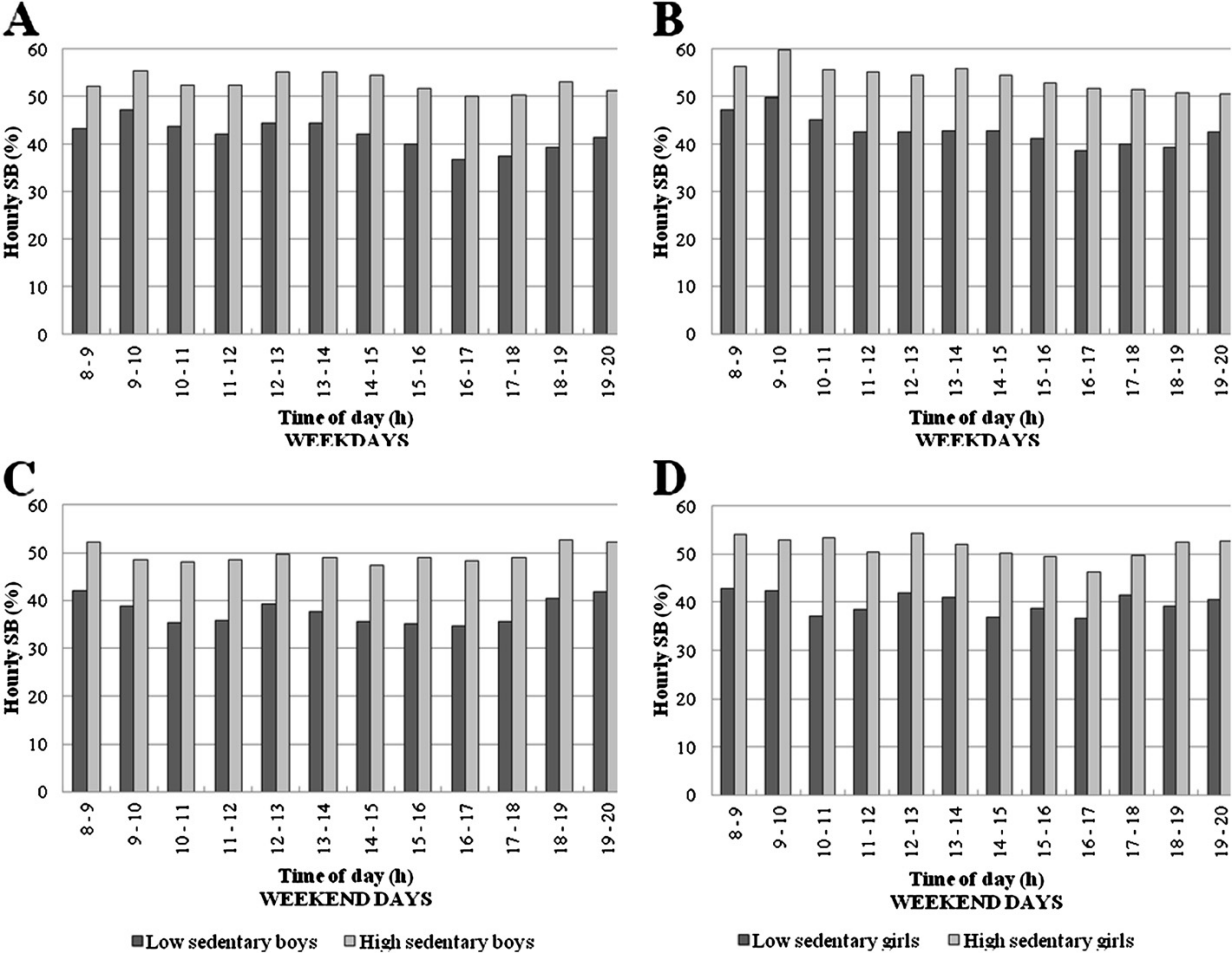
SB patterns hour-by-hour during weekdays and weekend days for boys and girls by SB level.

Hour-by-hour patterns of the percentage of time in SB during weekdays significantly differed between boys with low and high SB levels (χ^2^ (Δ 11 df) = 36.2). More specifically, differences in SB between the least and the most sedentary boys became greater from 14:00 h to 19:00 h (Figure 2A). In girls during weekdays (χ^2^ (Δ 11 df) = 10.3; p = 0.50; Figure 2B) and in boys (χ^2^ (Δ 11 df) = 8.3; p = 0.68; Figure 2C) and girls (χ^2^ (Δ 11 df) = 14.3; p = 0.22; Figure 2D) during weekends, no interaction effect was found with SB level. Further, substantially higher percentages of time per hour in SB were seen across the whole day in the most sedentary boys and girls compared to the least sedentary boys and girls (Figure 2A-D).

### SB patterns by weight status

**Figure 3:**
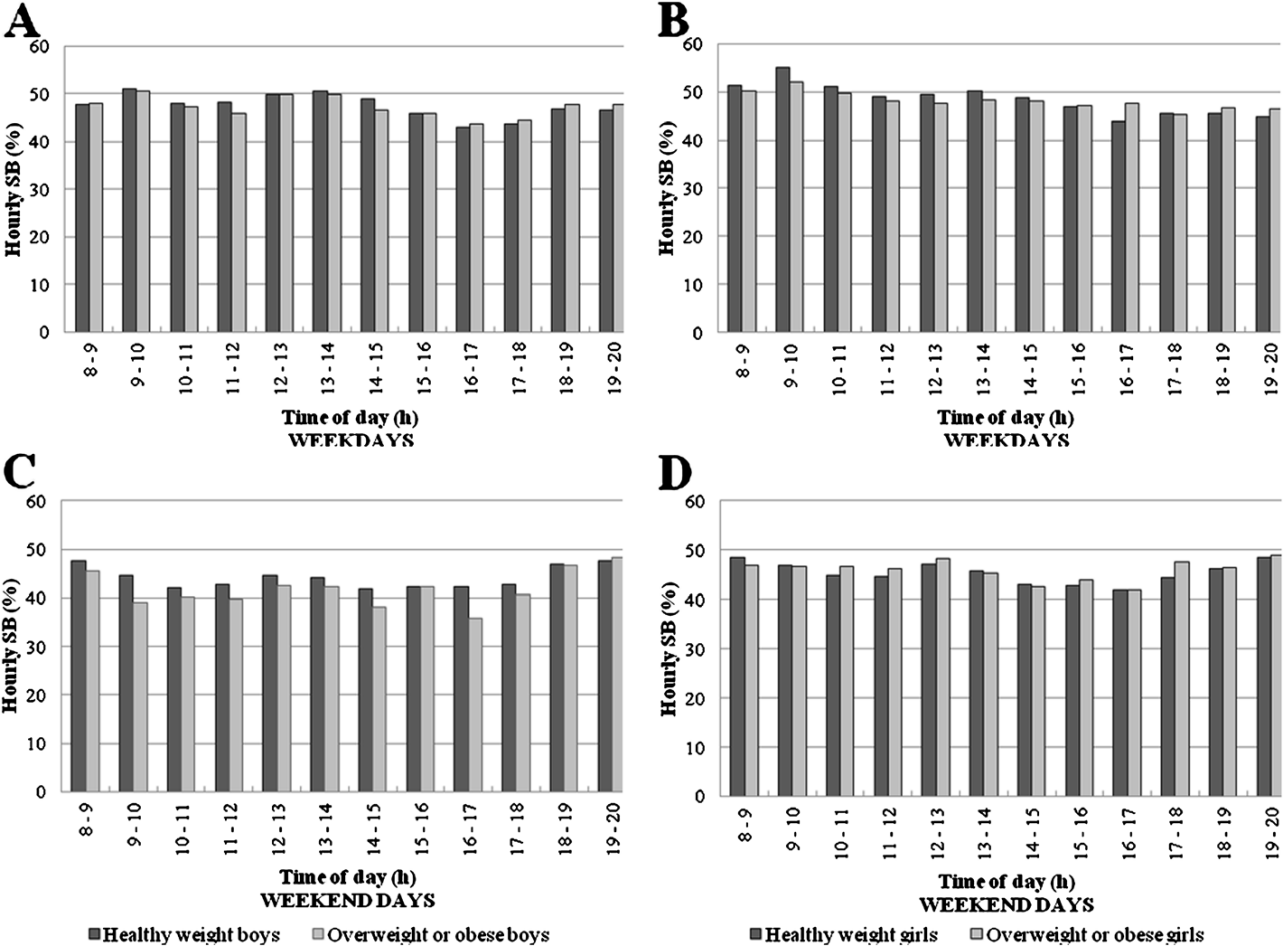
SB patterns hour-by-hour during weekdays and weekend days for boys and girls by weight status.

In boys and girls during weekdays (χ^2^ (Δ 11 df) = 9.5; p = 0.57 and χ^2^ (Δ 11 df) = 16.2; p = 0.14; Figure 3A and 3B, respectively) and during weekend days (χ^2^ (Δ 11 df) = 13.7; p = 0.25 and χ^2^ (Δ 11 df) = 5.3; p = 0.91; Figure 3C and 3D, respectively), the hour-by-hour patterns of percentage of time in SB were similar in healthy weight and overweight/obese children. Both groups also demonstrated similar percentages of time per hour in SB across the whole weekday and weekend day (Figure 3A-D).

### MVPA patterns hour-by-hour

**Figure 4:**
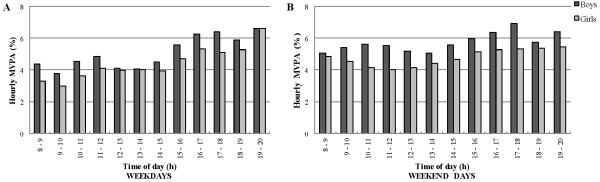
MVPA patterns hour-by-hour during weekdays and weekend days for boys and girls.

During weekdays, the percentage of time in MVPA varied significantly hour-by-hour in boys (χ^2^ (Δ 11 df) = 475.7) and in girls (χ^2^ (Δ 11 df) = 448.7) (Figure 4A). In boys, the lowest percentages of time per hour in MVPA were found from 09:00 h to 10:00 h and 12:00 h to 14:00 h. Percentage of time per hour in MVPA initially peaked from 10:00 h to 12:00 h in boys. After 15:00 h in boys, percentage of time in MVPA increased until the end of the day. Percentages of time in MVPA in boys from 15:00 h till 20:00 h were higher than the percentages between 08:00 h and 15:00 h. In girls, the lowest percentages of time per hour in MVPA were seen from 08:00 h to 10:00 h. After 10:00 h, the percentage of time per hour in MVPA increased until 12:00 h and then remained consistent until 15:00 h. After 15:00 h in girls, percentage of time per hour in MVPA increased again until the end of the day and the percentages between 15:00 h and 20:00 h were all higher than the percentages between 08:00 h and 15:00 h. From 08:00 h to 12:00 h and 14:00 h to 18:00 h during weekdays, girls were significantly more likely to exhibit lower percentages of time per hour in MVPA than boys (OR ranging from 0.75 to 0.87).

During weekend days, the percentage of time in MVPA varied significantly hour-by-hour in boys (χ^2^ (Δ 11 df) = 48.8) and in girls (χ^2^ (Δ 11 df) = 36.6) (Figure 4B). From 08:00 h till 15:00 h, percentages of time per hour in MVPA remained consistent and were the lowest throughout the day in both boys and girls. From 15:00 h to 20:00 h, percentages of time per hour in MVPA were the highest throughout the day in boys and girls. In boys, percentages of time per hour in MVPA increased after 15:00 h until 18:00 h and then dropped down a little bit until the end of the day. In girls, percentages of time per hour in MVPA between 15:00 h and 20:00 h remained consistent. From 08:00 h to 12:00 h and 14:00 h to 18:00 h, girls were significantly more likely to show lower percentages of time per hour in MVPA than boys (OR ranging from 0.75 to 0.88).

Differences in MVPA between weekdays and weekends were also seen. In boys, percentages of time per hour in MVPA from 09:00 h to 15:00 h during weekend days were significantly more likely to be higher compared to weekdays (OR ranging from 1.15 to 1.40). In girls, percentages of time per hour in MVPA from 08:00 h to 10:00 h and 14:00 h to 15:00 h during weekend days were significantly more likely to be higher compared to weekdays (OR ranging from 1.21 to 1.50) while the percentage of time per hour in MVPA between 19:00 h and 20:00 h during weekend days was significantly more likely to be lower compared to weekdays (OR = 0.87).

### MVPA patterns by low and high MVPA levels

**Figure 5:**
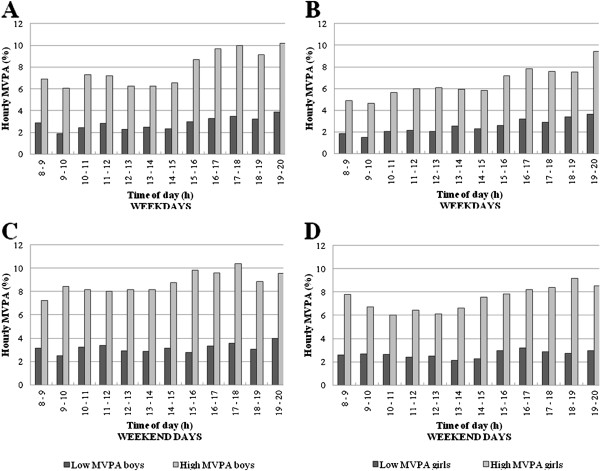
MVPA patterns hour-by-hour during weekdays and weekend days for boys and girls by MVPA level.

During weekdays and weekend days, the hour-by-hour patterns of percentage of time in MVPA did not differ between boys (χ^2^ (Δ 11 df) = 12.7; p = 0.31 and χ^2^ (Δ 11 df) = 13.8; p = 0.25; Figure 5 and 5C, respectively) and girls (χ^2^ (Δ 11 df) = 12.8; p = 0.31 and χ^2^ (Δ 11 df) = 9.2; p = 0.61; Figure 5B and 5D, respectively) with low and high MVPA levels. Lower percentages of time per hour in MVPA across the whole day were observed in boys and girls with low MVPA levels compared to boys and girls with high MVPA levels (Figure 5A-D).

### MVPA patterns by weight status

**Figure 6:**
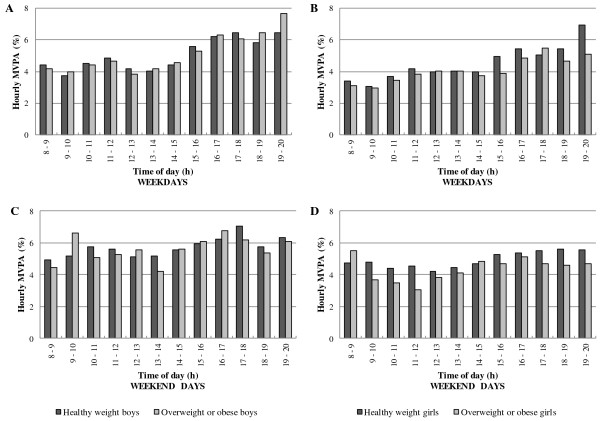
MVPA patterns hour-by-hour during weekdays and weekend days for boys and girls by weight status.

In boys and girls during weekdays (χ^2^ (Δ 11 df) = 8.5; p = 0.67 and χ^2^ (Δ 11 df) = 18.1; p = 0.08; Figure 6A and 6B, respectively) and during weekends (χ^2^ (Δ 11 df) = 9.4; p = 0.58 and χ^2^ (Δ 11 df) = 9.4; p = 0.58; Figure 6C and 6D, respectively), the hour-by-hour patterns of percentage of time in MVPA were similar in healthy weight and overweight/obese children. Percentages of time per hour in MVPA were also similar in both groups across the whole weekday and weekend day (Figure 6A-D).

## Discussion

The hour-by-hour analyses revealed that from the morning till the early afternoon on weekdays and in the morning and around midday on weekends were time periods where boys and girls have the highest amounts of SB and the lowest levels of MVPA. Besides, participation in SB was the lowest and participation in MVPA was the highest in boys and girls from the mid afternoon till the evening on weekdays and on weekends. The current findings also suggest that SB and MVPA could be improved throughout the whole weekday and weekend day as the variability across the included hours was quite small, especially in MVPA. Although during some hours, differences were found between boys and girls in SB and MVPA, differences were small in real time and indicate that there is no particular time of the week where one sex should be targeted. This study also indicated that weekdays offer the greatest opportunity to improve SB and MVPA as more favorable SB and MVPA levels per hour were found across weekend days compared to weekdays. Further, afternoon hours during weekdays were identified as a key period for the most sedentary boys as the differences between the least and the most sedentary boys in SB increased during these hours. Moreover, investigating the patterns by SB and MVPA level showed that the hour-by-hour SB levels were substantially higher in the most sedentary preschoolers compared to the least sedentary preschoolers while the differences in hour-by-hour MVPA levels between preschoolers with low and high MVPA levels were less pronounced. Finally, the results indicated that there was no particular time of the weekday and weekend day that would be more suitable to target either healthy weight or overweight/obese preschoolers.

A first key finding was that during weekdays, the time in SB was highest and participation in MVPA was lowest from the morning till the early afternoon in boys and girls. This time period during the less flexible weekdays could be characterized by essential daily living activities (e.g., bathing, dressing, or eating) or parents and childcare/preschool staff offering more sedentary activities to the children rather than active behaviors (e.g., watching TV, colouring, or being strapped into a car seat/buggy/chair), to enable them to prepare for the forthcoming daily tasks, to undertake household tasks, or to run errands. Furthermore, within the Australian context, children’s outdoor activity opportunities around the middle of the day are likely to be reduced to minimize sun exposure [43]. Conversely, from the afternoon till the evening on weekdays was identified as an opportunity for preschool children to engage in more active behaviors. Perhaps these time periods are associated with more free play time at preschool/childcare or at home. Other possible mechanisms are that children with older siblings would have then someone to play with at home, parents went with their child to the park, or children participated in some organized activities. These findings cumulatively highlight an intervention opportunity from the morning till the early afternoon on weekdays in boys and girls at the child care/preschool and home setting. Besides these identified time periods with the highest SB and lowest MVPA levels, the present findings suggest that the entire weekday should be targeted as the differences across the included hours in SB and especially in MVPA were rather small. This suggestion aligns with the recommended 180 minutes of PA per day for preschoolers that should be accumulated across the day rather than in one long session [5-7] and is supported by evidence from motor learning studies, showing that distributed practice conditions are superior to massed practice conditions for skill acquisition [44].

In contrast to our results, studies in older children and one study in preschool children, showed several marked peaks and troughs in hour-by-hour PA patterns on weekdays, representing closely the structured school day with sedentary classroom time and active breaks [20,45,46]. Following the end of the school day, one study reported increases [46] while others reported decreases in PA [20,45]. The differences between these patterns and those described in our study could be attributed to a high variability in the frequency and duration of attendance of our participants at childcare/preschool. Further, the daily program of the Australian childcare centers/preschools is less characterized by a structured curriculum and it can be expected that children receiving care from their (grand)parents do not follow a structured program throughout the weekday, hence distinct peaks and troughs in SB and MVPA are less likely.

Hour-by-hour patterns of SB and MVPA were less variable during weekend days than during weekdays, probably representing the higher flexibility for parents and children or more consistent engagement by parents with their children during weekends. The peaks in SB in boys and girls in the morning, around midday, and in the evening on weekends likely coincide with essential daily living activities (e.g., breakfast-, lunch-, and dinner-time, sedentary family time such as playing a game or watching television/a movie together, or preparing the children for bed-time). Further, the hour-by-hour patterns illustrated that on weekend days preschool children were also more active in the afternoon than in the morning. This increased activity can for example embody that families were active with their child or the child participated in organized activities. Thus, these findings indicate that SB and MVPA could be targeted in the morning, around midday, and in the evening on weekend days. Considering the current guidelines [5-7], the evidence from motor learning studies [44], the underlying mechanisms for these patterns, and the finding that the variability across the hours in SB and especially in MVPA was rather small during weekends, it may be more realistic to suggest to parents/carers to minimize time in SB and maximize the opportunities to be physically active for their preschool child in general and continually throughout the entire weekend day. Other studies in older children [45,46] and one study in preschool children [20], have also reported more consistent hour-by-hour activity patterns on weekends. In support of our results, Verbestel et al.’s study [20], involving 213 Belgian preschoolers, reported that average hour-by-hour accelerometer counts per minute during weekends increased significantly after 15:00 h and lasted till 18:00 h.

The current findings also suggest that there was no particular time of the weekday and weekend day where one sex should be targeted. This is in line with the findings from Verbestel et al.’s study in Belgian preschoolers [20]. Furthermore, it was established that preschoolers’ SB and MVPA were more favorable across the weekend days compared to the weekdays. Again, this can probably be explained by parents and children having more flexibility to engage in physical activities during weekends compared to weekdays. This observation highlights that weekdays, including its contexts and settings, are important intervention opportunities and future studies should examine which contexts and environments during weekends support lower levels of SB and higher amounts of MVPA. In addition, this finding might indicate that it is more difficult to improve SB and MVPA levels on weekdays compared to weekend days. In contrast to this study, most other studies in preschool children reported no differences in PA levels between weekdays and weekend days [14,17-21]. It is possible that these studies did not find differences because they only examined daily total PA rather than hour-by-hour PA intensities.

Studying the differences in the hour-by-hour patterns by SB and MVPA level indicated that afternoons during weekdays are an important intervention opportunity for the most sedentary boys. It appears that during these hours the most sedentary boys chose to participate in or are provided with less active activities than the least sedentary boys. Some evidence was also provided that to reduce SB, the most sedentary preschoolers, with substantially higher SB levels than the least sedentary preschoolers, are an important target group. To improve MVPA, all preschoolers despite their MVPA level should be targeted as relatively small differences were found between preschoolers with low and high MVPA levels. In the Avon Longitudinal Study of Parents and Children, it was shown that during weekdays the most active boys and girls were substantially more active during the period from the end of school to bedtime while during weekends the most active boys and girls showed peaks of activity during late morning and mid afternoon while the least active children exhibited extremely flat activity profiles [45]. However, data reported by Riddoch et al. [45] were for older children (mean age 11 yr) and patterns of hour-by-hour accelerometer counts per minute were examined, thus direct comparisons should be treated with caution. Finally, the present study also demonstrated that hour-by-hour patterns and levels of SB and MVPA during weekdays and weekend days did not differ when preschoolers were categorized into weight status groups, supporting the findings of two recently conducted reviews in preschool children where no association between BMI and SB [27] and BMI and PA was found [12].

Several strengths of the present study are noteworthy. The large, heterogeneous sample [28,29] is unique for accelerometer-based PA research in preschool children. In addition, the large sample size enabled us to stratify the data by type of day, sex, SB and MVPA level, and weight status, which has previously not been done. Further, using accelerometers allowed us to objectively report patterns of SB and MVPA hour-by-hour. Describing PA intensities instead of accelerometer counts per minute provided more meaningful and interpretable data.

The following limitations should be considered when interpreting our results. Although the sample size was large, the sample is not representative of Australian preschool children, particularly those who live in rural or regional areas. Furthermore, the generalisability of the present findings is limited by the low response rate of our study population (11% of the contacted parents agreed from the 46% of childcare centers/preschools participating). Previous studies in preschool children collecting accelerometry data for several consecutive days reported a childcare center/preschool participation rate between 50% and 100% [14,15,20,21] and a parental response rate between 43% and 67% [15,17,18]. Despite these higher response rates, the sample size (57 to 244 preschool children) was substantially lower in these studies compared to our study. The GT1M ActiGraph accelerometer has established validity in preschoolers, but some activities, such as swimming, bicycling, sitting, and standing cannot be captured [10,11,47]. Further, there is a lack of consensus on the most suitable cut points to use to classify accelerometer activity counts into PA intensity categories [11]. Although the investigation of patterns hour-by-hour is a strength of this study, it may be that studying SB and MVPA patterns hour-by-hour aggregated from all included weekdays and weekend days is still too crude. For example, aggregating data from all Saturdays and Sundays to investigate weekend patterns may mask potential differences between Saturdays and Sundays. Furthermore, the present study did not adjust for the environment in which children spent their time on weekdays and weekends, for example at home, with grandparents, or at childcare/preschool. Moreover, specific activities undertaken during time in SB and MVPA were not considered. Complementing accelerometer data with contextual information could provide more insights in which contexts and settings low/high levels of SB and low/high levels of MVPA are typical.

## Conclusions

In summary, entire weekdays, especially from the morning till the early afternoon, and entire weekend days are opportunities to reduce SB and to promote MVPA in preschool-aged boys and girls. Particularly weekdays appear to be important days of the week to improve both behaviors. To reduce SB, the most sedentary preschoolers are an important target group while to increase MVPA, all children despite their MVPA level should be targeted. No particular time of the week was found where one sex or weight status group should be targeted.

## Abbreviations

PA: Physical activity; SB: Sedentary behavior; MVPA: Moderate-to-vigorous physical activity.

## Competing interests

The authors declare that they have no competing interests.

## Authors' contributions

All authors were involved in the development of the research questions and the design of the study. TH, DC, and TO collected the data. EVC conducted data manipulation and analyses and drafted the manuscript. All authors contributed to the interpretation of data and were involved in the writing and critically revising of the manuscript. All authors read and approved the final version.
